# Their Influence upon Methods of Amputation

**Published:** 1917-11-24

**Authors:** 


### MODERN ARTIFICIAL LIMBS.

### Their Influence upon Methods of Amputation.

The ordinary meeting of the Medical Society of London on October 22 was devoted to a most instructive demonstration and discussion on the degree to which the recent advances in the construction of artificial limbs to minimise the war's ravages determine the particular form of amputation to be carried out. The chair was occupied by the President, Sir St. Clair Thomson, F.R.C.S. Major R. C. Elmslie, R.A.M.C. (T.), exhibited a number of patients to illustrate the main forms of apparatus, their uses, and limitations, and Mr. Muirhead Little, F.R.C.S., discussed the general question, and gave an epidiascope exhibition of photographs and skiagrams.

### The Function of an Artificial Limb.

Major Elmslie explained that practically all the limbs being shown were the products of Queen Mary's Convalescent Auxiliary Hospitals, Roehampton House. Before the establishment of that centre the supply of artificial limbs in the country to meet war results was quite inadequate, and many of those available were very elementary. There were now 100 varieties to meet various needs. The function of an artificial limb for the lower extremity was, primarily, to give stability in standing, and a reasonably good walking gait. There were two ways of taking a bearing from the body on to the artificial limb. First, by an end-bearing; secondly, by a bearing upon the bony surfaces above the site of the amputation. Another variety of bearing was that of the closely-fitting stump bucket, but it had the disadvantage that it pressed the skin upwards, and so drew it tightly across the cut end of the bone. The stump should fit closely in the bucket to within an inch of the lower end of the stump. The suspension of the artificial limb was carried out by means of shoulder-straps secured by leather thongs. When pressure was made on the bucket of the limb, if the line of what might be termed the centre of gravity lay just behind the centre of movement of the knee-joint, the limb would be stable, but if in front of the joint the limb would give way. The foot should be mounted in a position of slight equinus, *i.e.* pointing down a little in plantar flexion, so that when the limb met the ground the knee was forced into a slightly hyperextended position, and the limb was thereby rendered stable. The material mostly used for the construction of the limbs was willow, the bucket and leg being hollowed out of a single piece; the joint mechanism was steel, and the joints were bushed with a layer of leather, to restrict the joint movements. There were two outstanding problems to be solved in artificial limbs for the lower extremity: that of material and that of knee control. He showed a limb which was constructed of duralumin. In this material all the portions of a limb could be made in stock parts, and these could be fastened in place as required by a few metal clips. With regard to knee control, if the patient's weight came on the knee in the flexed position the knee would give way and the man would fall down. A leg had been invented which would give safety in any position, but this had not yet passed the experimental stage.

### The Artificial Arm.

The artificial arm must be regarded as a tool needed by the man in his employment. It was therefore desirable to have these implements in sections, so that they could be assembled at will for any special need. In this connection he showed an impressive array of fittings which the men wielded with great facility, including a sledge-hammer and a shovel.

### The Ideal Amputation Stump.

Mr. E. Muirhead Little said: The artificial hand of Gotz von Berlichingen, made in 1509, was still in existence and in working order; and it was said that its owner could strike a harder sword-stroke with it than with his former natural hand. To secure good results, the surgeon and the limb-maker must have some knowledge of each other's limitations. In this country, until the war, questions connected with amputations had been of diminishing importance, thanks to Lister, the Employers' Liability Acts, etc., so that Mr. Corner, of St. Thomas's, was able to state that in 1913, out of 5,483 major operations at that hospital, there were only 34 amputations, or 1 in 161. The Royal Surgical Aid Society supplied, in 1913, 529 artificial and peg legs and arms out of 41,483 appliances in that year. The conditions of the problem differed in the upper as compared with the lower extremity. The functions of the latter were crude when contrasted with the delicate and nicely co-ordinated movements of the upper extremity. The best artificial arm and hand was but a poor substitute for the living member, but an artificial leg could be made to do nearly everything required of its predecessor. The weight was borne (*a*) by direct pressure of a part of the body downwards, such as the end of the stump or of a convenient bony process, such as the tuberosity of the ischium; (*b*) by oblique pressure, when the socket formed a more or less conical cavity into which a conical portion of the body fitted. These two were often combined. Leverage was exerted by the lateral surface of the stump pressing against the side of the surrounding socket. The ideal amputation stump should be as long as possible, and covered by sound, movable soft parts, the scar being placed in a position where it would not be exposed to pressure; nor be adherent to bone. It should have enough muscles attached to it to move it with adequate force, and there should be no inflamed or hypersensitive nerve ends palpable in it. In addition, the end or ends of the bone or bones should be rounded, so as not to hurt the soft parts under them, the blood supply free, and the skin well nourished.

**MODERN ARTIFICIAL LIMBS**—(*continued*).

What and When to Avoid.

Experience of modern artificial limbs had led to the conclusion that, except in the cases of the hip and shoulder, exarticulations were to be avoided, and that amputation through the limb segment above the joint was preferable. He thought the following objections against amputations through joints were valid: (1) The articular enlargement made the resulting stump bulbous in form, and this became more marked as atrophy of muscles progressed, leaving the dimensions of the rigid bone unaltered. This could not be fitted with a rigid bucket, such as formed the best support. (2) The artificial joint must either be fitted below its proper level, or must be placed on the sides of the bone end, and the limb must be still wider than its fellow, and consequently unsightly. (3) To cover the articular enlargement, extensive flaps were needed, and sometimes these must consist of thin skin; hence they were often deficient in vitality and liable to slough. In the lower extremity, a stump capable of bearing part of the body weight on its end should be aimed at. Of 549 cases of amputation of the lower extremity seen recently at Roehampton, it was found advisable to fit an end-bearing pad in 275, or one-half, though the proportion varied widely in the different regions, being only 9 per cent. in the upper third of the thigh, in the middle third 32 per cent., and in the lower third 77 per cent. The general conclusion was that the stumps in the lower third of the thigh were much more sound than those in the upper and middle thirds. In the upper extremity the question of end pressure did not arise. Here the condition of the lower part of the anterior and the external surfaces of the stump was of most importance, because these surfaces have to take much of the pressure of the leverage action of the bucket. The presence of osteophytes was scarcely any hindrance; Mr. Little showed skiagrams of some cases in which these occurred.

The Choice of Situation.

Detailed consideration was devoted to the different sites of amputations. The objections to amputations through joints in general applied with special force to the elbow, owing to the shape of the bones here. With regard to the shoulder region, the action of the biceps tendon, which tended to throw off the bucket when it contracted in flexion, was one of the chief difficulties in short forearm stumps. But the tendon could not be spared, so its removal was out of the question. But the remains of the bellies of the muscles had been removed in some cases, with more or less advantage, thus making possible a better grip on the stump. The lower third of the forearm was not a favourable site; the stumps were often cold and cyanotic, the skin lightly stretched and adherent, and sometimes the bone ends were tightly soldered together by fibrous tissue, and pronation and supination power were lost. With regard to below-the-wrist cases, if the carpus and the base of the metacarpus could be saved, that was worth while, provided movement was preserved. The short stump resulting could be used to actuate a movable thumb with considerable force by means of a suitable appliance.

Thigh and Leg Joints.

Mr. Little said he did not hesitate to affirm emphatically that in the lower limb, from an anatomical point of view, exarticulation or amputation between the trochanters was better than amputation in the upper third of the thigh, as this, in the average limb, included a little more than six inches of femur, thus giving only three or four inches of stump below the pubes. Such a short stump could not move the lever formed by the thigh bucket with sufficient force, no matter how light the limb. Many patients preferred the "tilting table" to the ordinary thigh bucket, and he regarded it as one of the greatest improvements in artificial limb-making during the war. One of the greatest troubles of the orthopædic surgeon and the limb-maker was due to the presence of flexion contraction of the hip in cases of thigh amputation. It was natural that the primary consideration should be the early and complete healing of the wound. To secure this, and for the comfort of the patient, the stump was often propped up upon a pillow, thus favouring shortening of the hip flexors. This contraction, unless severe, was marked by a compensatory lordosis. Practically every case admitted to Queen Mary's Hospital had some flexion-contraction or a diminution of the normal range of hyperextension, and a course of massage and passive motion, or tenotomy or fasciotomy, had to be carried out before a leg could be satisfactorily fitted. In the leg below the knee, the most favourable site was the lower part of the middle third. The old seat of election, about four inches below the joint, was intended for kneeling legs. A good gait had been obtained with as little as 2½ inches of tibia, though this was an exceptional case. Usually at least three inches was required, and then the stump should be capable of end-bearing. Much depended on the bulk of the soft parts, for when they were voluminous the difficulty was increased. Amputation in the lower third of the leg seldom gave a satisfactory stump, and here Symes' operation was deserving of its great reputation, as, if properly done, it gave a painless weight-bearing stump. The bones should be sawn through well above the internal malleolus, so as to avoid too bulbous a stump, and care taken to make the section at right angles to the long axis of the whole leg, not at right angles to the often incurved lower fourth. Chopart's amputation gave a good bearing surface, but generally the calf muscles pulled up the heel and tilted the lower end downwards, causing painful pressure on the scar.

In reply to a question, Mr. Little stated that an artificial leg usually lasted about seven years, but generally needed some attention at the end of eighteen months. Patients varied much in the reception they gave an artificial arm; some did not wish for them, whilst others said they could not do without them.

